# The Double Face of Base Excision Repair: Preventing and Triggering Double‐Strand Breaks

**DOI:** 10.1002/bies.70092

**Published:** 2025-11-21

**Authors:** Susan M. Gasser

**Affiliations:** ^1^ Université de Lausanne ISREC Foundation Agora Cancer Research Center Lausanne Switzerland

## Abstract

How cells repair oxidative damage to DNA has been studied for over 60 years. Recent evidence confirms that the base excision repair (BER) machinery not only acts to restore an intact double DNA helix by replacing oxidized bases, but under some circumstances, BER goes awry, generating double‐strand breaks and provoking chromosome fragmentation. This fragmentation can lead to extensive genomic rearrangements that correlate with oncogenesis. Whether the BER factors suppress or promote DNA damage depends on multiple parameters: the nature of the damage, the clustering of modified bases, the pathway of BER chosen, and chromatin remodelers. Recent data leading to this unexpected role for BER are reviewed here.

## Graphical abstract

Base excision repair is conserved from bacteria to man, robustly eliminating oxidized bases in DNA and reinserting the appropriate nucleotide to restore an intact double helix. Recent results describe conditions and mechanisms that misregulate long‐patch BER to generate double‐strand breaks, triggering chromosome fragmentation and cell death, altering BER's classic role.

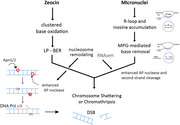

## 1 Introduction

Two aspects of eukaryotic genome stability are widely accepted. First is the fact that double‐strand breaks (DSB) are the most deleterious DNA lesions found in dividing cells [1]. The second is that base oxidation is the most common DNA lesion incurred, as it arises from internal metabolic processes and is enhanced by environmental stressors (i.e., UV, ionizing radiations, pollutants, and heavy metals) and xenobiotics (i.e., antiblastic drugs) [2]. This review will examine links between the two types of damage.

Although there are multiple pathways for DSB repair, notably non‐homologous end‐joining (NHEJ), micro‐homology mediated end‐joining (MMEJ) and homologous recombination (HR), the repair of a DSB brings the risk of sequence insertion, deletion, frameshift, inversion, as well as long DNA damage‐induced checkpoint arrests and/or chromosomal translocations that alter genome structure [1]. These latter can generate circular or dicentric chromosomes, which perpetuate the cycle of breakage and repair, typical for a cancer cell. When cells override the DNA damage checkpoint at G2/M triggered by a DSB, chromosome fusions or circular chromosomes are often mis‐segregated and end up in micronuclei [3]. If a damaged cell, on the other hand, fails to divide and reverts back to interphase, aneuploidy and polyploidy ensue, states found in ∼90% of human tumors [4]. Given this risk, it is not surprising that one finds a multitude of factors that either carry out or control DSB repair among tumor suppressor genes, the so‐called “guardians of our genome” [5]. These include genes for the repair factors BRCA1, BRCA2, PALB2, p53, MSH2, and MSH6, as well as the relevant checkpoint kinases ATM, ATR, CHEK2, and all subunits of the Mre11‐Rad50‐Nbs1 complex, and the RecQ helicases BLM and WRN. Genes encoding the HR proteins RAD51C and RAD51D are also frequently found mutated in cancer, even if they are not formally tumor suppressors [6].

**FIGURE 1:**
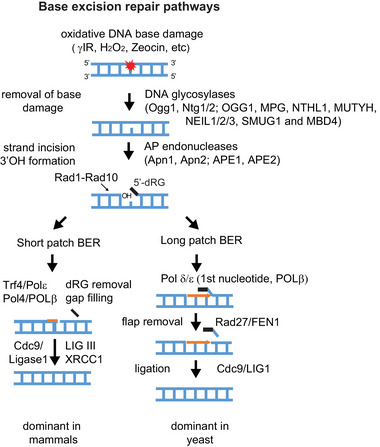
The two pathways of base excision repair. Scheme of short‐patch (SP) and long‐patch (LP) BER pathways with yeast enzymes in lower case and mammalian enzymes in capitals. In SP‐BER the equivalence of Pol4 and POLβ is not entirely clear and the polymerase Trf4 may play a more major role in yeast. The human glycosylases are numerous, and this may not be an exhaustive list. We note that APE1 (also known as APEX1, HAP1, or Ref‐1) is not the only enzyme that processes 3′ ends to generate a 3′OH, but in humans, polynucleotide phosphatase/kinase (PNKP) can do so as well. For long‐patch repair DNA pol β may insert the first nucleotide (Figure reproduced from Ref. [34]).

It is equally well‐documented that an oxidative intracellular environment is oncogenic [7, 8]. Reactive oxygen species (ROS) damage nucleotide bases, amino acids, and lipids and promote angiogenesis by stimulating growth factors, cytokines, and transcription factors like VEGF and HiF‐1α [9, 10, 11]. We note that base oxidation is a common occurrence even under normal cell growth, as it can arise from intrinsic metabolism, including mitochondrial respiration, peroxisomal metabolism, and enzymatic activities like that of cytochrome P450 in the endoplasmic reticulum and of NADPH oxidases in the cytosol. To deal with both endogenous and pathological ROS, cells contain highly efficient base excision repair (BER) pathways that replace oxidized bases, abasic sites and bases with small adducts in DNA at breakneck speed throughout the cell cycle without triggering the DNA damage checkpoint [9, 12, 13, 14, 15]. Tens of thousands of oxidized bases are repaired per cell per day, through excision of the modified base, cleavage of the sugar backbone upstream of the abasic site, followed by repair of the single‐stranded gap by one of two well‐conserved pathways (Figure 1). In mammalian cells, the most common gap‐filling mechanism is called short‐patch repair, mediated by DNA Pol β, which fills in 1–2 nucleotides, followed by ligation by a dedicated complex of XRCC1 and Ligase 3. There is an alternative long‐patch BER pathway, which is dominant in yeast, during which a longer stretch of DNA is synthesized by DNA pol δ or ε from a 3′ OH often generated by APE1/Apn1, the most abundant abasic site endonuclease [14]. The displaced flap is cleaved by FEN1 (known as Rad27 in budding yeast), after which Ligase 1 seals the nicked double helix (Figure 1). Both DNA BER pathways are conserved from yeast to man [13, 16], although the preferred choice pathway for BER can vary. Exactly what determines the choice in a given species is largely unknown [11, 17].

### 1.1 Base Excision Repair as a Guardian of the Genome

BER not only repairs alkylation and oxidative damage caused by endogenous and exogenous ROS, but it also recognizes inosine and other noncanonical nucleotides as single‐base lesions [18, 19]. These include *N*‐alkylated purines (*N3*‐methyladenine, *N7*‐methylguanine, and *N3*‐methylguanine), 8‐oxo‐7,8‐dihydroguanine(8‐oxoG), thymine glycols, 5‐OH and 6‐OH dihydrothymine, uracil glycol, 5‐hydroxycytosine, urea residues, and deoxyinosine. The diversity of lesions can be accommodated in BER thanks to a range of DNA glycosylases (Figure 1) that recognize and excise damaged or aberrant bases selectively, all generating an apurinic or apyrimidinic (AP) site, while leaving the sugar‐phosphate backbone of the DNA helix intact [20]. Most DNA glycosylases are monofunctional (e.g., *N*‐methyl purine DNA glycosylase [MPG]) and simply excise the damaged base [21]. There are also bifunctional glycosylases like OGG1 and NTHL1, which can both cleave the sugar linkage to the base and nick the phosphodiester backbone, 3′ to the AP site [22, 23, 24]. Lesion type determines engagement of the more relevant enzyme; for example, NTHL1 is involved in the repair of pyrimidines, such as thymine glycol, while OGG1 acts primarily on the mutagenic lesion 8‐oxo‐guanine and cytotoxic Fapy‐G [25]. If a base is removed without cleavage of the sugar backbone, APE1 recognizes the abasic site and cleaves the DNA backbone, creating a reparable single‐strand (ss) nick [20]. APE1 is encoded by *APEX1* in humans (*APN1* in yeast) and is the most abundant DNA endonuclease in most cells [14, 26, 27]. The human APE2 (encoded by *APEX2*) is closely related, but is found in a distinct multicomponent complex. Both endonucleases are tightly regulated in order to avoid inappropriate nicking of the DNA backbone, which, if not rapidly repaired, could engender a DSB [11, 14].

A synthetic lethality screen performed by the Elledge laboratory [28] supports the notion that BER (and AP endonucleases) are “guardians of the genome,” cleaning up base lesions or single‐strand nicks before either the transcription or replication machineries convert the lesion into a DSB. Their screen was aimed at finding pathways that render cancer cells tolerant to a BRCA2 mutant that lacks its DNA‐binding domain, compromising HR. The screen was not done under conditions of augmented stress (i.e., cells were not exposed to radiation or oxidizing chemicals), but in normally dividing human colon cancer and/or ovarian cell lines under standard cell culture conditions (which are, however, hyperoxic) [28, 29]. They found synthetic lethality with the *BRCA2* mutant upon ablation of *POLQ* (DNA polymerase θ), *FEN1*, or *APEX2*. The recovery of *POLQ* (DNA Pol θ) as a CRISPR hit was interpreted as demonstrating an elevated requirement for micro‐homology mediated end‐joining (MMEJ), given that MMEJ, which supports DSB repair in cells deficient for HR, requires this error‐prone  DNA polymerase. Another hit was FEN1, the “flap endonuclease,” which participates in both MMEJ and long‐patch BER. To explain the impact of *FEN1* and *APEX2* ablation, the authors argue that by compromising BER, cell death is enhanced in HR‐deficient cells by increasing the occurrence of DSBs. This is in contrast to the loss of MMEJ, which acts in parallel to HR as a redundant DSB repair pathway. The authors propose that the loss of BER acts upstream of HR, and that by compromising BER the number of ss lesions that become DSBs increases. In conclusion, interference in either BER or MMEJ repair pathways are synthetic lethal with the loss of HR (i.e., partial *BRCA2* loss of function), for different reasons.

In this context it is interesting to note that *APEX1* and *XRCC1*, both key enzymes of short‐patch BER, were only weakly synthetic lethal with the *BRCA2* allele, in contrast to *APEX2*. Unlike APE1, which binds Pol β and XRCC1 [14], APE2 is in complex with PCNA. PCNA is not needed for the XRCC1‐dependent short‐patch BER pathway (Figure 1) [13], as DNA pol β is non‐processive [30, 31]. Instead, PCNA is engaged in long‐patch BER, where it acts with DNA pol ε of δ, and sometimes FEN1 [19]. A plausible interpretation of these results is that the selective loss of long‐patch BER increases DSB frequency to overwhelm the *BRCA2*‐deficient cell [28]. Although it is surprising that short‐patch BER scored less strongly, these results are consistent with conventional wisdom, which implicates BER in protecting the genome from DSBs by repairing ss lesions. Consistently, DNA damage checkpoint kinases were exceptionally active in the *APEX2‐BRCA2* double mutant [28].

### 1.2 BER Can Actively Generate DSBs

In contrast to this study, two recent papers describe a surprising gain‐of‐function lethality for long‐patch BER, showing that the rapid fragmentation of chromosomal DNA actually depends on enzymes of the BER pathway [32, 33, 34, 35]. In other words, in these studies, BER enzymes (most notably APE1/APE2 in human or Apn1/Apn2 in yeast) generated DSBs, rather than preventing them, as argued in the Elledge paper. Both Shimada et al. and Tang et al. document a massive and relatively rapid fragmentation of the genome that is called chromothripsis [36, 37] in mammalian cells, and yeast chromosome shattering (YCS) [33] in budding yeast. Chromothripsis appears to occur primarily in micronuclei, a common feature of cancer cells [37, 38], and micronuclei arise when chromosomes or chromosome fragments fail to attach to the spindle, lag behind during anaphase, and get excluded from the reforming nucleus in a daughter cell. In micronuclei, these “forgotten” chromosomal fragments often undergo extensive genomic fragmentation and reassembly into a single composite chromatid that can then be propagated clonally among the cancer cells (reviewed in [36]). Although in some cases these events lead to cell death, the religated chromosomal fragments can generate fusion proteins that can drive oncogenesis. This is enhanced in dividing cells exposed to microtubule‐inhibiting agents, and is distinct from apoptosis [37, 38].

In pursuit of the mechanism leading to chromosome fragmentation, the Pellman laboratory found that extensive chromosomal fragmentation can stem from an accumulation of deoxyinosine in the DNA strand of RNA‐DNA stretches found in chromosomes in micronuclei [32]. In human cells, deoxyinosine is removed by a glycohydrolase, MPG, and the abasic site becomes a substrate for cleavage by the BER endonuclease APE1. Given that in such RNA:DNA hybrids or R‐loops the annealed RNA strand can be rapidly degraded by RNAseH [39], the action of APE1 at an abasic site in an R‐loop would generate a nick that would generate a DSB if the looped out single‐strand DNA were cleaved, for instance at the ds‐ss junction. Pellman argues that the tendency for micronuclei to rupture allows the preferential processing of micronuclear genomic fragments that are rich in deoxyinosine as compared with the rest of the genome. Micronuclei rupture may also increase access to a cytoplasmic pool of APE1, as well as RNAseH, both of which are tightly controlled in the nucleus. Rupture plus the disproportionate accumulation of RNA‐DNA hybrids and/or deoxyinosine in micronuclei may explain why micronuclear DNA accumulates DSB and succumbs to chromothripsis [37].

In parallel to studies in mammalian cells, a somewhat similar and massive fragmentation of budding yeast chromosomes into fragments of 50–100 kb (called YCS for Yeast Chromosome Shattering) has been reported for wild‐type yeast cells exposed to low doses of Zeocin, a base‐oxidizing antibiotic, along with non‐toxic levels of a TORC2 inhibitor [33]. Cytoplasmic actin filament depolymerization and nucleosome remodeler deregulation are at the root of the TORC2 inhibitor effect in combination with base‐oxidation [35] (to be reviewed elsewhere). Relevant to this review is the fact that base oxidation by Zeocin, at a level that is easily tolerated in cells with an intact cytoskeleton, triggers conversion of ss lesions to DSBs upon F‐actin depolymerization [33, 34]. The fragmentation occurs rapidly, without passage through the cell cycle, is independent of apoptotic mechanisms, and does not reflect a loss of HR or NHEJ pathways of DSB repair [33, 34]. On the other hand, it is heavily dependent on the AP endonucleases Apn1 and Apn2, on N‐glycosylases and the downstream polymerases of long‐patch BER [33, 34].

Modeling shows that roughly 100 irreparable DSBs per genome would be necessary to fully fragment the haploid genome of budding yeast into 100‐kb fragments [34]. Upstream of this massive DSB induction is base oxidation by bleomycin‐like agents, or less efficiently, by exposure to γ irradiation (γIR). Intriguingly, H_2_O_2_, which also oxidizes bases, but in a completely random fashion, does not trigger YCS [34]. The lesions generated by all these agents are N‐glycosylase substrates, most frequently 8‐oxo‐G [9, 10]. The processing of 8‐oxo‐G generates an apurinic (AP) site that allows the endonucleases (Apn1, Apn2, and Rad1/Rad10, in yeast) to nick the DNA backbone, which is then repaired by short‐ or long‐patch BER (Figure 1). In yeast long‐patch BER is the dominant repair pathway and is capable of repairing 1000s of base oxidation events throughout the cell cycle (Figure 1). In mammalian cells the dominant pathway is short‐patch BER; the parameters that determine pathway choice are unclear, although yeast lacks XRCC1 and Ligase 3, and has an atypical DNA Pol β [9, 11, 15].

### 1.3 How Can Low Levels of Base Oxidation Generate DSBs?

**FIGURE 2:**
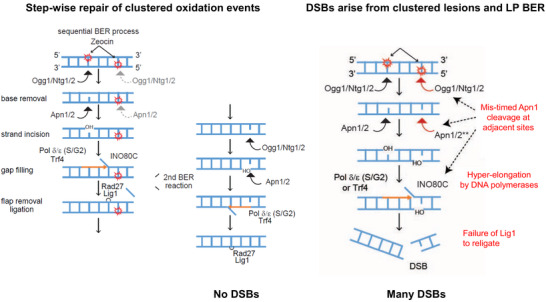
How cells deal with clustered oxidative lesions and nicks: normal and pathological outcomes of BER. The repair of clustered oxidization events on DNA can become toxic, generating DSBs, due to a miscoordination of the two‐step BER process. The normal step‐wise repair of two closely positioned oxidized bases on opposite strands is shown on the left. In budding yeast, upon inhibition of TORC2 [33], or disruption of cytoplasmic actin polymerization [35], there is a premature processing of the second lesion by AP endonucleases and/or the accelerated elongation of second strand synthesis from the first lesion by DNA Pol δ (in S phase) or by Trf4 and DNA pol ε. Both events can generate a DSB from two closely juxtaposed oxidative lesions. We note that only yeast enzymes are named here for simplicity. Mammalian names are indicated in Figure 1. Modified from Ref. [34].

Early work by Povirk and colleagues showed that bleomycin and related antibiotics have the unusual property of inducing closely positioned oxidative damage on opposite strands of the paired double helix, and it is this clustering of damage that—in one case out of ten—generates a DSB [10, 40, 41]. In DNA exposed to bleomycin (or Zeocin, which is a bleomycin derivative), ss nicks outnumber DSBs by 10 to 1, whereas after γIR the ratio is 100 to 1. In other words, the formation of a DSB, arising from clustered oxidation events, occurs 10 times more frequently “per base oxidation” (or ss nick) after exposure to bleomycin versus γIR in vitro [10, 24]. Beyond that, in living cells the processing of closely juxtaposed oxidation events on opposite strands must take place in a sequential manner, in order to avoid DSB generation [17, 42, 43]. In other words, untimely cleavage by the AP endonuclease at the second lesion, prior to the ligation of the first, can generate a DSB from two relatively innocuous oxidized bases on opposite strands (Figure 2). By testing mutants in the BER pathway for YCS, Shimada showed that the fragmentation of yeast chromosomes is driven by long‐patch BER and may be relevant for lesions separated by as much a kb of intact DNA [34]. Regulating both the Apn1‐mediated cleavage of adjacent abasic sites on opposite strands and polymerization of the new strand, are crucial for successful long‐patch BER [34, 35].

The genetic analysis of YCS showed that both N‐glycosylases and AP nucleases are implicated in the generation of DSBs in S‐ and G2‐phase yeast cells, whereas in G1‐arrested cells the loss of AP nucleases, but not N‐glycosylases, blocked fragmentation [34]. This suggests that AP endonuclease access and/or activity is rate‐limiting for repair. The conversion of juxtaposed base oxidation lesions into breaks was also influenced by altering elongation rates of repair polymerases, and was increased by the activation of an actin‐containing nucleosome remodeler that enhances polymerase processivity [34, 35]. While in yeast this was dependent on the INO80C remodeler complex [35], SWI/SNF may be the relevant complex in mammalian cells [44]. It is well established that nucleosomes can block the action of early BER enzymes [45, 46, 47], thus hyperactivation of INO80C may allow inappropriate access of the second lesion to Apn1 (Figure 2). In conclusion, if two adjacent DNA lesions are processed in a sequential manner, with the second cleavage occurring after the ligation of the first, the risk of DSB induction is low. However, when long‐patch BER polymerases and/or Apn1 activity are upregulated, which can be triggered by increased levels of nuclear G‐actin [35], then sequential processing fails and chromosomes tend to shatter.

### 1.4 The Role Played by AP Endonucleases

It is clear that AP endonucleases, encoded by *APN1* and *APN2* in yeast and *APEX1* and *APEX2* in mammals, have complex roles in the response to DNA damage [14, 48]. In mammals, not only does APE1 cut at apurinic or apyrimidinic sites generated by glycosylases that excise damaged bases, but together with Thymine DNA glycosylase (TDG), APE1 catalyzes the first steps in BER‐mediated demethylation of cytosines. In this context, APE1 also triggers the release of TDG and other glycosylases from an abasic site. Mammalian APE1, like yeast Apn1/Apn2, also participates in what is called the BER/NIR switch [49]. In this latter, APE1 or Apn1/Apn2 bypasses the action of the glycosylase and generates a ss nick that retains a 5′ damaged nucleotide and generates a 3′OH group [50]. The APE1 switch from acting downstream of a glycosylase to a glycosylase‐independent mode appears to depend on allosteric changes in APE1 structure, potentially controlled by its N‐terminal tail [51]. It is possible that during YCS, Apn1/Apn2 is acting in this glycosylase‐independent mode.

**FIGURE 3:**
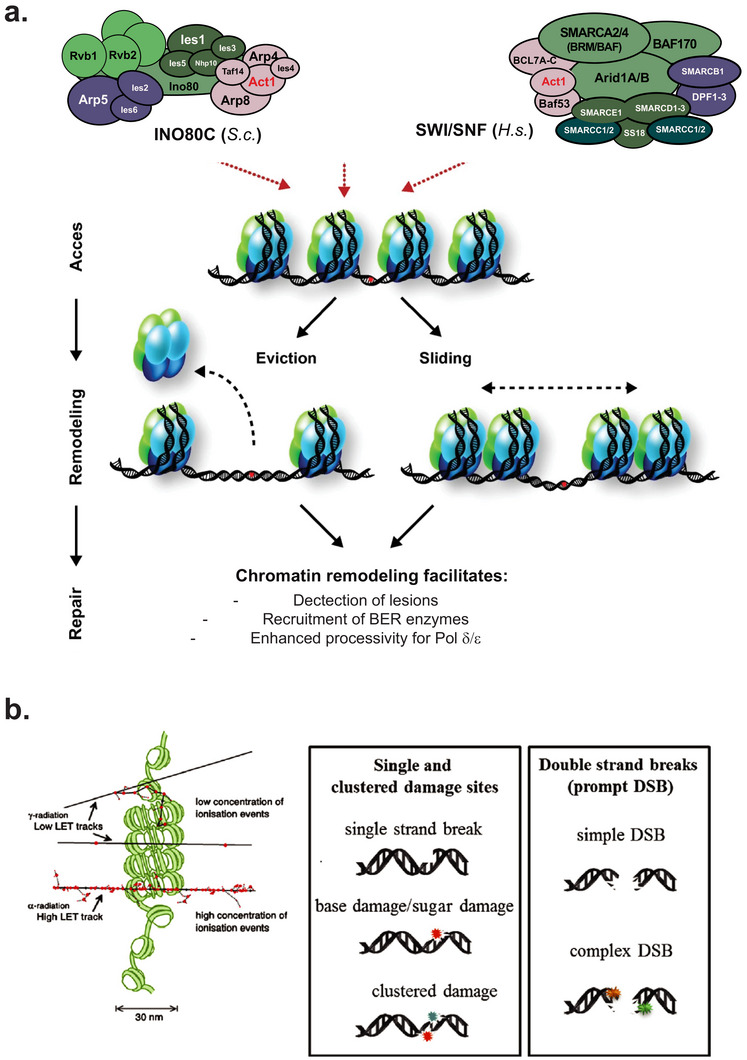
The impact of chromatin on DNA damage and repair. (a) Subunit composition of the *S. cerevisiae* INO80C and the *Homo sapiens* BAF/BRG or SWI/SNF nucleosome remodelers. Each contains actin as a core subunit in complex with actin‐like proteins (Arp4 or BAF53), a rate‐limiting component for remodeling activity, which may explain the impact of actin depolymerization on remodeler function [35]. Subunit names are indicated, but we note that SWI/SNF complexes come with multiple different subunit compositions and that the major subunits have multiple names. The sketch of the impact of nucleosome remodelers is from Ref. [59]. The fact that nucleosomes can block BER enzymes is documented in Refs. [45, 46, 47]. (b) This sketch from Lomax et al. (Ref. [55]) shows schematically the potential impact of chromatin compaction of the types of damage provoked by low and high ionizing radiation beams (LET = linear energy transfer). Damage can range from single‐strand base modifications that lead to ss nicks, generating clustered lesions of which some may occur on opposite strands. Their processing may generate a DSB, as described above. DSBs can either be simple (easily ligated, usually after lesion processing by the upstream BER enzymes) or complex, requiring other end‐processing enzymes to generate appropriate donor and acceptor sites.

We imagine two mechanisms that lead to Apn1/APE1 hyperactivation. One depends on an aberrant increase in nuclear Apn1/APE1 levels, given that this appears to be a key element in controlling AP endonuclease activity. Consistently, overexpression of Apn1 fused to a nuclear localization signal rendered yeast cells hypersensitive to Zeocin [34]. Alternatively, the AP endonucleases may simply have enhanced access to lesions, as in the case of RNA‐DNA hybrids, thanks to activated nucleosome remodelers [52] (Figure 3a). This is particularly relevant as nucleosomes can block BER [45, 46, 47, 52], and thereby may regulate access to the second Apn1 cleavage in clustered lesions (Figure 3a).

The control of BER by nucleosome remodeling is an important parameter that requires further study [45, 46, 47, 52]. Nonetheless, a priori, it clear that by enhancing nucleosome removal or shifting nucleosomal position, Apn1/APE1 access to a second, adjacent lesion is enhanced, and that if the lesions are found on opposite strands, this can generate a DSB [34, 35]. Furthermore, hyperextension by DNA pol δ, which is known to be regulated by INO80C [53], can generate a DSB if, during long‐patch BER, this highly processive polymerase encounters a nick on the opposite strand. In addition, Apn1 and Apn2 both possess 3′ to 5′ exonuclease activity [54], which means that a misregulated AP nuclease could resect a DNA strand until it encounters a nick on the other strand, again generating a DSB (Figure 2). Such aberrations may occur less frequently in mammalian cells than in yeast, given that mammalian cells rely preferentially on short‐patch BER, yet conditions likely exist in mammalian cells that favor long‐patch BER or else upregulate APE1/APE2 activity and DNA pol δ (or possibly DNA pol β) processivity. In this context it is also important to explore the mechanisms that control the choice between short‐ and long‐patch BER [11].

### 1.5 Clinically Relevant Implications of BER Misregulation

γ‐irradiation oxidizes bases in DNA much like Zeocin or bleomycin do, although lesions are generally thought to be randomly distributed in the genome [10, 24, 40]. This would account for Povirk's measurement that ss nicks are 100 times more frequent than DSBs after IR. On the other hand, if DNA is complexed with histones and assembled into a higher folded structure, a single irradiation beam, such as those used to treat cancer patients, could generate clustered base oxidation events on a single helix [55] (Figure 3b). We predict that this, coupled with an inhibitor of short‐batch BER enzymes (e.g., Ligase 3), would increase the lethality of radiation therapy by generating clustered lesions.

Early work from the Wallace laboratory explored the potential for BER enzymes to convert IR‐induced damage into DSBs [25, 56]. They found that clustered lesions, which occur more frequently in irradiated cells than in cells treated with H_2_O_2_, are often converted into DSBs, and that this depends on the level of OGG1 and NTHL1 glycosylases [25, 56]. Overexpression of these bifunctional glycosylases enhanced DSB formation, yet they did not test DSB formation following bleomycin or Zeocin. On the other hand, it was shown in human HCT116 cells that lethal DNA breaks can be induced by Zeocin if F‐actin is depolymerized, an observation that parallels results in yeast [35, 57]. If the observations that implicate long‐patch BER in YCS can be extended to human cells, it is conceivable that the lethality of IR‐induced damage could be augmented by activating AP endonucleases, bifunctional glycosylases, and/or DNA pol δ processivity in long‐patch BER (see, e.g., [58, 59]). Triggering this by nucleosome remodeler upregulation is therapeutically also an option, although these observations remain to be tested in a clinical setting.

While it is counterintuitive to think that upregulated BER generates, rather than prevents DNBs (as argued in [28]), it should be noted that the conditions under which the loss of *APEX2* was synthetic lethal with *BRCA2* deficiency, were not equivalent to the situations in which solid tumors are exposed to IR. Living up to its reputation as a “double‐edged sword” [25], we propose that it will be possible to upregulate APE1 activity, or OGG1 or NTHL1 activities, to convert clustered base oxidation lesions into DSBs in mammalian cells. For the moment, those events appear to be restricted to the abundant R‐loops found in micronuclei, but as the internal cellular states that regulate BER pathways are identified, we may find ways to both prevent and foster double‐strand breaks in genomic DNA as well. We conclude that BER, and in particular APE1/Apn1, can cut both ways with respect to genome integrity, both generating and preventing DSBs. Understanding the factors that shift this balance may be key to improving clinically relevant radiation treatments, through combination or adjuvant therapies.

## Conflicts of Interest

The authors declare no conflicts of interest.

## Data Availability

Data sharing is not applicable to this review as no datasets were generated or analysed during generation of this commentary.
